# Potential biological therapies for severe preeclampsia: a systematic review and meta-analysis

**DOI:** 10.1186/s12884-019-2268-9

**Published:** 2019-05-09

**Authors:** Sophia Grimes, Kira Bombay, Andrea Lanes, Mark Walker, Daniel J. Corsi

**Affiliations:** 1 OMNI Research Group, Clinical Epidemiology Program, Ottawa Hospital Research Institute, Ottawa, Ontario Canada; 2 School of Epidemiology, Public Health and Preventive Medicine, University of Ottawa, Ottawa, Ontario Canada; 3 Department of Obstetrics, Gynecology & Newborn Care, University of Ottawa, Ottawa, Ontario Canada; 4 Children’s Hospital of Eastern Ontario Research Institute, Ottawa, Ontario Canada; 5 OMNI Research Group, Centre for Practice Changing Research, Ottawa Hospital Research Institute, L1242, Box 241, 501 Smyth Road, Ottawa, ON K1H 8L6 Canada

**Keywords:** Preeclampsia, Mesenchymal stem cells, Antithrombin, Alpha-1-microglobulin, Gestational hypertension, Systematic review, Meta-analysis

## Abstract

**Background:**

Preeclampsia remains a significant danger to both mother and child and current prevention and treatment management strategies are limited. The objective of this systematic review was to investigate the current literature on evidence for the use of the regenerative capacity of mesenchymal stem cell (MSC) therapy, the anticoagulant activity of antithrombin (AT), or the free radical scavenging activity of alpha-1-microglobulin (A1M) as potential novel treatments for severe preeclampsia and Hemolysis, Elevated Liver enzymes, Low Platelet count (HELLP).

**Method:**

We conducted a systematic review of potential biological therapies for preeclampsia. We screened MEDLINE and Embase from inception through May 2017 for studies using AT, A1M or MSCs as potential treatments for preeclampsia and/or HELLP. A meta-analysis was performed to pool data from randomized control trials (RCTs) with homogenous outcomes using the inverse variance method. The Newcastle-Ottawa Scale, the Cochrane risk of bias tool for RCTs, and SYRCLE’s risk of bias tool for animal studies were used to investigate potential bias of studies.

**Results:**

The literature search retrieved a total of 1015 articles, however, only 17 studies met the selection criteria: AT (*n* = 9, 8 human and 1 animal); A1M (*n* = 4, 3 animal and 1 ex-vivo); and, MSCs (n = 4, 3 animal and 1 ex-vivo). A meta-analysis of AT therapy versus placebo and a meta-analysis for AT therapy with heparin versus heparin alone did not show significant differences between study groups. Animal and ex-vivo studies demonstrated significant benefits in relevant outcomes for A1M and MSCs versus control treatments. Most RCT studies were rated as having a low risk of bias across categories with some studies showing an unclear risk of bias in some categories. The two cohort studies both received a total of four out of nine stars (a rating of “poor” quality). Most animal studies had an unclear risk of bias across most categories, with some studies having a low risk of bias in some categories.

**Conclusions:**

The findings of this review are strengthened by rigorous systematic search and review of the literature. Results of our meta-analyses do not currently warrant further exploration of AT as a treatment of preeclampsia in human trials. Results of animal and ex-vivo studies of A1M and MSCs were encouraging and supportive of initiating human investigations.

**Electronic supplementary material:**

The online version of this article (10.1186/s12884-019-2268-9) contains supplementary material, which is available to authorized users.

## Background

Preeclampsia (PE) is a condition that arises solely in pregnancy [1]. It is characterized by high blood pressure and proteinuria, and can have fatal consequences [1]. PE can become severe when the diastolic blood pressure reaches or exceeds 110 mmHg [1]. Ten percent of women have high blood pressure during pregnancy, and PE complicates between 2 and 8% of pregnancies [2]. Overall, 10 to 15% of direct maternal deaths are associated with PE and eclampsia [2]. Although the etiology of PE is not confirmed, there are numerous hypotheses related to disturbed placental function. Current research suggests that PE evolves in two pathophysiological stages [3]. In the first stage, poor spiral artery adaption due to decreased trophoblast ingrowth of the myometrial arteries leads to decidua-associated vascular changes, causing oxidative stress which damages the placenta [3]. As a result, there is uneven placental flow and reduced oxygen concentration [3]. The second stage of preeclampsia is associated with maternal endothelial dysfunction which precedes the onset of clinical manifestations including hypertension, proteinuria, and edema [3]. HELLP (hemolysis, elevated liver enzymes, and low platelet count) syndrome is serious for the mother and the fetus [4]. HELLP occurs in 0.2–0.8% of pregnancies and in 70–80% of cases it coexists with PE [4]. It is characterized by prominent endothelial damage in the liver [5]. The endothelial damage triggers an immune response inducing thrombotic microangiopathy with platelet-fibrin thrombi in micro-vessels [5]. The resulting angiopathy leads to consumption of circulating platelets, causes hemolysis in affected micro vessels and reduces portal flow in liver; leading to life threatening events [5].

The only confirmed treatment for PE and/or HELLP is induction of delivery, which often results in preterm birth [2]. In addition, babies that are born with low birthweight or congenital anomalies may have higher rates of coronary heart disease, high blood pressure, high cholesterol concentrations, and abnormal glucose-insulin metabolism [6]. There is ongoing research being performed on potential biological therapies for HELLP and severe PE. Three approaches that are currently being investigated include: exploiting either the anticoagulant activity of antithrombin (AT), the free radical scavenging activity of alpha-1-microglobulin (A1M), or the regenerative capacity of mesenchymal stem cells (MSCs) [7].

First, a decrease in AT levels has been seen in patients with PE [8]. Since the formation of thrombin is closely linked to inflammation through the thrombin receptors, vasoconstriction can be accompanied by a hypercoagulative state in PE [9]. Canonical correlation analyses have shown that the clinical severity shown in PE is related to hypercoagulativity [9]. Therefore, AT treatment is a possible treatment to improve maternal and fetal condition in PE [9].

Second, higher levels of cell-free fetal hemoglobin (HbF) have been found in placentas of patients diagnosed with PE [10]. HbF has been shown to have properties which promote inflammatory and oxidative tissue damage [11]. A1M has been identified as a reductase and binder of heme- groups and organic radicals [10]. It has properties that can protect cells and tissues in particular against damage from free Hb-, heme-, and oxidative stress [11]. Although increased concentration of A1M has been found in urine and plasma from women with PE, it is hypothesized that endogenously produced A1M cannot cope with the levels of HbF and oxidative stress which occur in PE [11]. Further, it has been suggested by Wester-Rosenlöf and colleagues that by intravenous supplementation of recombinant A1M can compensate for increased HbF and oxidative stress levels may relieve symptomatic clinical expression of PE [11].

Finally, MSCs are adult stem cells with self-renewal and multi lineage differentiation potentials [12]. Human MSCs have shown to exert antinflammatory, immunoregulatory and repair effects in animal models that include models of cardiac disease, lung injury and hypertension [12]. The bone marrow is the major source of MSCs [12]. However, the procedure to obtain MSCs from the bone marrow is invasive, and the number of MSCs reduces with age, limiting the clinical applications of MSCs [12, 13]. It has since been discovered that human umbilical cord blood is rich in MSCs that contain similar features to the MSCs found in bone marrow [12]. Therefore, the regenerative effects of placenta or umbilical cord-derived MSCs could potentially treat the endotoxin-induced hypertension that occurs in preeclamptic patients [12]. The objective of this study was to investigate the use of AT, A1M or MSC’s as potential therapies for the treatment of women with HELLP and/or PE using evidence from non-clinical and clinical studies.

## Methods

We conducted a systematic review of the studies done using AT, A1M or MSCs as potential treatments for severe PE or HELLP. The protocol for this systematic review was registered on PROSPERO International prospective register of systematic reviews (registration number: 42017069324) [14]. This study adheres to PRISMA reporting guidelines and methodology for systematic reviews and meta-analyses [15].

### Search strategy

A search strategy was developed using MESH terms and keywords related to the population and interventions. Ovid MEDLINE and Embase databases were searched from inception until May 2017 (see Additional file 1 for full search strategy).

### Study selection

Eligible studies included those with pregnant women who had been diagnosed with PE (high blood pressure and proteinuria), severe PE and/or HELLP syndrome. Studies that included pregnant animals who had been induced to develop preeclamptic or HELLP-like symptoms were also eligible, as well as ex-vivo or in-vitro models. Eligible studies for inclusion used one of the following treatments as the intervention: AT, A1M or MSCs (placebo or accepted therapies as comparators). There were no restrictions based on language of the study publication. One or more of the following outcomes were required in the study for it to be included: change in blood pressure, change in proteinuria, gestational week at delivery, or days of prolonged pregnancy. All titles and abstracts were independently screened by two reviewers using Covidence [16], and any inconsistencies were resolved through discussion. A full-text screen of the included studies was then performed. Studies were excluded if both reviewers determined that the articles were irrelevant or adequate translations were not achievable.

### Data abstraction and assessment of quality

Data were abstracted during the second full-text screen from the articles that met all inclusion and exclusion criteria. A data abstraction tool was independently used by both reviewers (SG and KB) to systematically extract information from the included studies. Study design, type of publication, population size, study objective, specific patient population, treatment used and outcomes included for each study and confounding variables adjusted for in the analysis. The Newcastle-Ottawa Scale, the Cochrane risk of bias tool for quality assessment of randomized controlled trials and SYRCLE’s risk of bias tool for animal studies were used to investigate potential bias of studies [17–19]. The Newcastle-Ottawa Scale was used to assess cohort and case-control studies, and assessed potential bias in the following categories: selection, comparability and outcome [17]. The Cochrane risk of bias tool was used for randomized control trials (RCTs) and the bias was assessed according to the guidelines as good, fair, or poor quality [18]. The SYRCLE’s risk of bias tool was used for animal studies [19]. The inter-observer variability between reviewers for assessing bias was done using Kappa statistics. Six questions were selected from different parts of the data extraction form including; funding source, industry involvement, type of publication, study design, exposure and intervention. The average kappa statistic for these six questions was 0.81.

### Data synthesis and analysis

A minimum of two RCTs with homogenous outcomes were required to perform a meta-analysis. Where studies included subjects satisfying similar selection criteria, presenting similar outcomes, and defining measurements in a same manner, the effect sizes were synthesized in a fixed effect model. The inverse variance approach was used for continuous data, with standard mean difference as the effect measure. The I^2 statistic along with chi-squared and degrees of freedom were computed to check for heterogeneity among studies included. The test for the overall effect of the model was noted to be statistically significant at *p* < 0.05.

Data from RCTs, observational studies, animal studies, and ex vivo studies were also captured in summary Tables.

## Results

**Fig. 1:**
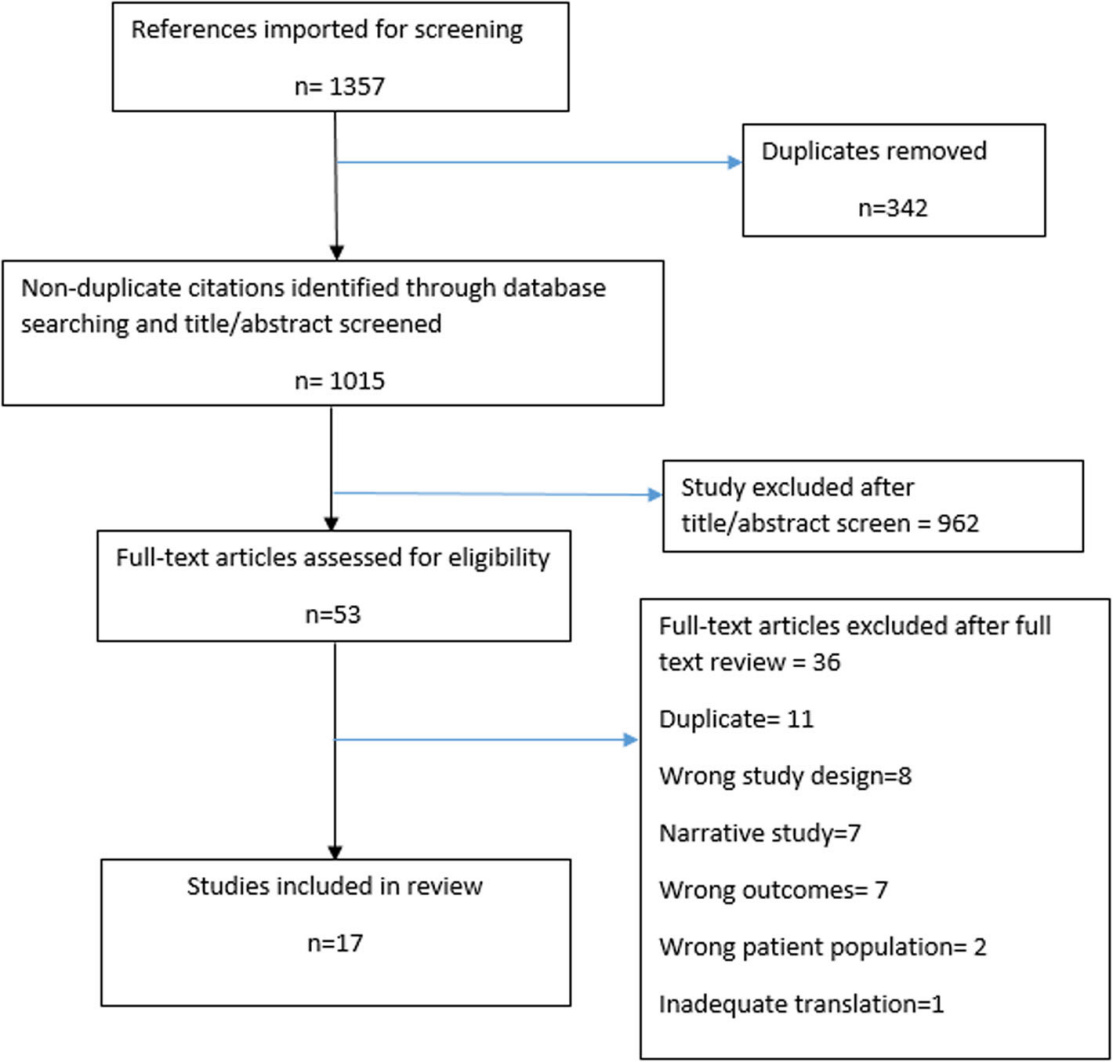
Preferred Reporting Items for Systematic Reviews and Meta-Analyses (PRISMA) flow diagram showing study selection process

Database searches identified 1015 non-duplicate references imported for screening. Their title and abstracts were screened and 962 studies were excluded from the primary screen, which resulted in 53 studies remaining to undergo full-text screening (Fig. 1). After the full-text screen, 17 articles remained in the study. Of these 17 studies, nine investigated the use of AT as the treatment (six RCTs [9, 20–24]), two observational studies [25, 26], and one animal study (reported in two publications [27, 28]). Four investigated the use of A1M as treatment (three animal studies [11, 29, 30] and one ex vivo study [31]), and four investigated the use of MSCs as treatment (three animal studies [12, 32, 33] and one ex vivo study [34]). All human and animal studies looked at the effect of these treatments on PE or induced PE-like symptoms. None of the studies investigated HELLP syndrome.

Of the 17 studies, nine studies were published since 2014, four studies had a publication date between 2000 and 2013, and the remaining four studies had a publication date prior to 2000. Forty-seven percent of the studies were from Asia, 41% of the studies were from Europe, and 12% were from North America. Forty-seven percent of the studies were animal studies, 35% of the studies were RCTs, 12% were cohort studies, and 12% were ex-vivo studies.

The most commonly investigated outcome was level of proteinuria, which was an outcome mentioned in six animal studies [12, 27, 29, 30, 32, 33] and two human studies [22, 24]. All seven animal studies [11, 12, 27, 29, 30, 32, 33] and one human study reported on blood pressure values [26], six human studies reported gestational age at delivery [9, 20, 18, 23, 24, 26], and five human studies reported on prolongation of pregnancy from treatment to delivery [20, 22–25].

### Assessment of quality

The Cochrane risk of bias tool was used to assess RCTs [18]. Fifty percent of the studies were rated as having an unclear risk of bias for the sequence generation category, with 50% being rated as having a low risk of bias (Table 1). The same results were obtained for allocation concealment, as well as blinding of participants and personnel. Eighty-three percent of the studies were rated with a low risk of bias for the incomplete outcome data assessed category, with one study (17%) being rated with an unclear risk of bias. The same results were obtained for the free of selective reporting category. One hundred percent of the studies were rated with a low risk of bias in the free of other bias category.

**Table 1:** Results of assessment of risk of bias for Randomized Control Trials, based on Cochrane risk-of-bias-tool [18]

Reference	Sequence generation	Allocation concealment	Blinding of participants and personnel	Blinding of outcome assessments	Incomplete outcome data assessed	Free of selective reporting	Free of other bias
D’Angelo, 2016 [22]	Low	Low	Low	Low	Low	Low	Low
Kobayashi, 2003 [21]	Unclear	Unclear	Unclear	Low	Low	Low	Low
Maki, 2000 [20]	Unclear	Unclear	Low	Low	Low	Low	Low
Sameshima, 2008 [9]	Low	Low	Low	Low	Low	Low	Low
Sibai, 2017 [23]	Unclear	Unclear	Unclear	Unclear	Low	Unclear	Low
Paternoster, 2004 [24]	Low	Low	Unclear	Low	Unclear	Low	Low

The Newcastle-Ottawa Scale [17] was used twice for quality assessment on the two cohort studies [25, 26]. These studies received an average of 3 out of 4 stars for selection, 0 out of 2 stars for comparability, and 1 out of 3 stars for outcome. They both received 5 out of 9 stars total.

The SYRCLE risk of bias tool was used to assess the animal studies [19]. It should be noted that the one animal study which investigation AT was reported in two publications [27, 28]; both are assessed here and the higher quality report [28] was summarized in the results below. Most or all studies were rated as low risk of bias for the group similarities at baseline category, the free of selective reporting category, and the free of other bias category; one was rated as having a high risk of bias (free of selective reporting category), while the rest were rated as unclear (Table 2). Most studies were rated as unclear for the sequence generation category, the allocation concealment category, the random housing of animals’ category, and the blinding of caregivers/investigators and outcome assessor category; the rest were rated as low risk of bias (Table 2). Over half the studies were rated as low risk of bias for the random animal selection category, while the rest were rated as unclear (Table 2). Over half the studies were rated as low risk of bias for the outcome assessment category, while the rest were rated as unclear (Table 2).

**Table 2:** Results of assessment of bias for animal studies, based on SYRCLE risk-of-bias tool [19]

Reference	Sequence generation	Group similarities at baseline	Allocation concealment	Random housing of animals	Blinding of caregivers and/or investigators and outcome assessor	Random animal selection for outcome assessment	Incomplete outcome data addressed	Free of selective reporting	Free of other bias
Erlandsson, 2014 [29]	Unclear	Low	Unclear	Unclear	Unclear	Low	Unclear	Low	Low
Fu, 2015 [12]	Low	Low	Unclear	Low	Unclear	Low	Low	Low	Low
Liu, 2014 [35]	Unclear	Low	Unclear	Low	Unclear	Low	Unclear	Low	Low
Naav, 2015 [30]	Unclear	Unclear	Unclear	Unclear	Unclear	Low	High	Unclear	Low
Shinyama, 1996 [28]	Unclear	Low	Unclear	Unclear	Unclear	Unclear	Low	Low	Low
Wang, 2016 [33]	Unclear	Low	Unclear	Unclear	Unclear	Unclear	Low	Unclear	Low
Wester-Rosenlof, 2014 [11]	Low	Low	Low	Unclear	Low	Low	Low	Low	Low

### Antithrombin

For studies evaluating AT, the population, dose and outcomes reported for the studies are presented in Table 3. Eight of the nine studies studied pregnant women with PE [9, 20–26], with the remaining study was conducted in a rat model of PE [28]. The eight human studies varied in their study populations since the inclusion criteria between studies varied with regards to gestational age. The total gestational age range covered in these studies was 23 to 35 weeks. Three human studies used purely AT as the treatment and placebo as the control [22, 23, 25]. Two human studies used AT alone as the treatment and human albumin as the control [9, 20]. One human study looked at high dose AT versus a low dose of AT [24]. The remaining two human studies used a combination of AT and heparin as treatment and heparin alone as the control [21, 26]. The animal study had three study groups, low-dose AT, high-dose AT, and placebo [28].

**Table 3:** AT Outcome Table

Reference	Population	Dose		Blood pressure (mmHg)		Proteinuria (g/day)		Gestational age at delivery (weeks)		Pregnancy prolongation (days)	
		AT	Control	AT	Control	AT	Control	AT	Control	AT	Control
D’Angelo, 2016 [22]	Pregnant women diagnosed with PE before 30WG (*n* = 38)	3000 IU/daily of AT for 7 days or less until delivery	Placebo, 1% glycine, for 7 days or less until delivery			Diagnosis: 2.28 ± 2.31 Pre-partum:4.02 ± 3.60	Diagnosis: 2.31 ± 1.68 Pre-partum: 4.58 ± 4.28			Median: 4.5 Range: 1–55	Median:3.5 Range: 1–57
Maki, 2000 [20]	Pregnant women diagnosed with PE between 24WG and 35WG (*n* = 133)	3000 IU/daily of AT for 7 days or less until delivery	Placebo, 582 mg human albumin, once daily for 7 days					34.1 ± 3.2**	33.0 ± 2.7	Prolonged gestation by 6.5 compared to placebo **	N/A
Sameshima, 2008 [9]	Pregnant women diagnosed with PE before 32WG (*n* = 82)	3000 IU/daily of AT for 7 days or less until delivery	Placebo, 582 mg human albumin, once daily for 7 days					24–27: *n* = 5 28–29: n = 2 30–31: *n* = 11 32–33: *n* = 10 34≤: *n* = 11* (compared to control at 34≤)	24–27: n = 1, 28–29: n = 11 30–31: n = 9 32–33: *n* = 16 34≤: *n* = 4		
Sibai, 2017 [23]	Pregnant women diagnosed with PE between 23WG and 30WG (*n* = 120)	rhAT 250 mg loading dose followed by a continuous infusion 2000 mg/24 h	Placebo, identical to AT dose except with saline					28.8 ± 2.7		11.2 ± 14.6	16.1 ± 20.9
Paternoster, 2007 [25]	Pregnant women diagnosed with PE between 24WG and 30WG (*n* = 88)	5000 IU on day 1, then received a dose that was calibrated to achieve a plasma level of a minimum of 120% of baseline value for the next 6 days.	Placebo							Unknown. States that pregnancy was prolonged in AT group.	N/A
Paternoster, 2004 [24]	Pregnant women diagnosed with PE between 24 and 33WG (*n* = 23)	Received one dose of AT sufficient to reach a plasma AT activity of at least 80% of the normal as well as 3000 U/day for 5 days	One dose of AT sufficient to reach a plasma AT activity of at least 80% of the normal			Baseline: 1.56 Change: 5.14	Baseline: 3.07 Change: 2.85	28.76 ± 3.83	29.35 ± 3.10	6 ± 2.1	3.5 ± 3.4
Nakabayashi, 1999 [26]	Pregnant women with PE before 32WG (*n* = 29)	1500 U/day of AT concentrate plus 5000 U/day of heparin was administered intravenously for 7 days	5000 U/day of heparin for 7 days	Systolic: 145.3 ± 3.2** Diastolic: 110.3 ± 4.9	Systolic: 161.4 ± 2.9 Diastolic: 106.1 ± 4.5			32.1 ± 2.5	31.8 ± 2.3		
Kobayashi, 2003 [21]	Pregnant women diagnosed with PE between 24WG and 36WG (n = 29)	1500 U/day of AT concentrate plus 5000 U/day of heparin was administered intravenously for 7 days	5000 U/day of heparin for 7 days					32.3 ± 3.1	29.8 ± 3.7		
Shinyama, 1996 [28]	Pregnant rats fed high salt diet (*n* = 36)	Low dose group: 60 U/kg/day for 10 days; High dose group: 300	Placebo, 3 mL/kg/day of saline for 10 days	High dose: 233*; Low dose: 236	At 15–17 DG: 249mmHg	High (300 U/kg/day): 32*; Low (60 U/kg/day): 51	At 17–19 DG: 93.5 (mg/kg/24h)				

*DG* day of gestation, *WG* week of gestation

*statistically significant at *p* < 0.05

**statistically significant at *p* < 0.01

For the five studies comparing AT versus placebo or human albumin, none of the studies reported values for blood pressure. However, one of the articles [20] includes a gestosis index which combines edema, proteinuria, and blood pressure to make a numerical scale that is used to compare the effectiveness of AT versus placebo on improving these three outcomes. The lower the gestosis index became, the more effective the treatment was. The gestosis index was significantly decreased in the AT treated group. In terms of proteinuria, only one of the studies [22] included numerical values, which did not change significantly with treatment. The other study that investigated proteinuria was Maki, 2000 [20] which incorporated proteinuria into the gestosis index. The gestosis index was significantly decreased in the AT treated group. Of the five studies that reported pregnancy prolongation as an outcome [20, 22–25], one study found that AT prolonged gestation significantly (6.5 days) compared with human albumin (*p* < 0.01) [20]. Another study reported that pregnancy was prolonged in the AT group compared with placebo, although it did not provide data supporting the statement [25]. The remaining three studies did not report any significant differences in pregnancy prolongation between the AT and control groups [22–24].

For the two studies comparing AT and heparin versus heparin alone in women with PE [21, 26], only one study reported blood pressure as a treatment outcome [26]. This study found that the group treated with AT and heparin had a significantly lower systolic blood pressure compared with the group treated with heparin alone (*p* < 0.01). Neither studies reported proteinuria values, however, proteinuria was incorporated into the gestosis index of one study [21], which reported that proteinuria was significantly improved in the AT plus heparin treated group. Both studies reported no significant differences in gestational age in the AT and heparin treated group compared with heparin alone.

For the single study that compared high-dose versus low-dose AT in women with PE, no significant differences in proteinuria, gestational age and pregnancy prolongation were observed between study groups [24].

Finally, for the animal study that compared high-dose AT, low-dose AT and placebo in a rat model of PE, a significant improvement in blood pressure and proteinuria was observed in the high-dose AT group (but not the low-dose AT group) compared with placebo [28].

### Alpha-1-microglobulin

For studies evaluating A1M, the population, dose and outcomes reported for the studies are presented in Table 4. The three studies investigating A1M treatment were all animal studies, and used different study animals: mice [29], rabbits [30] and ewes [11]. Various methods were used to achieve preeclamptic-like symptoms seen in pregnant women, such as using transgenetics, administering HbF infusions, or inducing starvation. However, in the study that used HbF, the blood pressure was not significantly increased to preeclamptic-like levels and therefore the effect of A1M on blood pressure could not be assessed. The treated groups were given different doses of A1M and all of the control groups used placebo. One study stated that the A1M treatment significantly reduced hypertension and proteinuria throughout pregnancy, though no data was given [29]. Another study showed that the mean arterial blood pressure was significantly lower (*p* < 0.05) than the control group [11]. The level of proteinuria was significantly reduced (p < 0.05) compared to the control in another study [30].

**Table 4:** A1M Outcome Table

Reference	Population	Dose		Blood pressure (mmHg)		Proteinuria	
		A1M	Control	A1M	Control	A1M	Control
Erlandsson, 2014 [29]	STOX1 transgene mouse model (n = NR)	A1M injections from DG6	Placebo, buffer solution	Unknown. States that A1M significantly reduced hypertension throughout pregnancy.	N/A	Unknown. States that A1M significantly reduced proteinuria throughout pregnancy.	N/A
Naav, 2015 [30]	Rabbits infused with 20 mg/kg of HbF (*n* = 19)	6 mg/kg of A1M	Nothing	HbF didn’t increase BP		11 μg/mL of albumin*	57 μg/mL of albumin
Wester-Rosenlof, 2014 [11]	Ewes starved for 36 h (n = 11)	2 bolus doses of 1.8 mg A1M/kg body weight 2 h apart	2 separate 50 mL doses of buffer solution	MAP 36 h post treatment: 58.5*	MAP 36 h post treatment: 73.5		

RED: Approximate value obtained by graph extrapolation

*MAP* mean arterial pressure, *DG* day of gestation, *BP* blood pressure

*statistically significant at *p* < 0.05

The one ex-vivo study investigating A1M used HbF to induce functional and structural damage to human term placentas such as extracellular vesicle release and placental microRNA (miRNA) expression alteration [31]. These changes are seen in PE and are hypothesized to be caused by toxic effects of free HbF [31]. A1M is thought to be able to counteract the effect that HbF has on the placentas [31]. Placental tissue was perfused ex-vivo with free HbF and the miRNA profile in released extracellular vesicles were examined along with the therapeutic effect of A1M. Free Hb altered the miRNA expression of placental extracellular vesicles. The up-regulated miRNAs; mir-373, mir-372 and mir-527 are all expressed on chromosome 19, close to the placental imprinted gene cluster C19MC [31]. Mir-424 is down-regulated in hypoxic trophoblasts [31]. A1M reduced the Hb induced changes, suggesting heme scavenging as a potential therapeutic approach in PE [31].

### Mesenchymal stem cells

For studies evaluating MSCs, the population, dose and outcomes reported for the studies are presented in Table 5. The three studies investigating MSC treatment were animal studies, with two studies using rats [12, 33] and one study using mice [32]. The treated groups were given different doses of MSCs and all of the control groups were untreated. In the two rat studies, collectively, there were 3 out of 6 days where the blood pressure and the proteinuria was significantly lower (*p* < 0.05) than their control counterparts [12, 33]. In the one mouse study, there were no significant differences in proteinuria, however, a significant lowering of systolic blood pressure was observed (*p* < 0.01) [35]. The one ex-vivo study investigating MSCs looked at the potential of placenta-derived MSCs to counteract the pro-inflammatory cytokines and anti-angiogenic factors typical of preeclamptic placentae [34]. This study treated PE villous explants with conditioned media collected from cultured placenta-derived MSCs isolated from control placentae. The results showed that normal placenta-derived MSCs conditioned media treatment significantly decreased Macrophage-migration Inhibitory Factor, Tumour Necrosis Factor α, Interleukin 6 and anti-angiogenic soluble fms-like tyrosine kinase-1 mRNA levels in PE villous explants relative to untreated controls [34].

**Table 5:** MSCs Outcome Table

Reference	Population	Dose		Blood pressure (mmHg)		Proteinuria	
		MSC	Control	MSC	Control	MSC	Control
Fu, 2015 [12]	Rats treated with 2 mL endotoxin (*n* = 21)	MSCs suspended in 100 μL phosphate-buffered saline (2 × 10^6^ cells/100 μL)	Nothing	Systolic DG8: 113 DG15: 124.5* DG19: 124.0*	Systolic DG8: 112 DG15: 131.0 DG19: 135.5	DG9: 3.15 mol/L DG16: 4.60 mol/L* DG20: 5.60 mol/L*	DG9: 3.20 mol/L DG16: 3.65 mol/L DG20: 3.90 mol/L
Liu, 2014 [32]	Mice injected with 10^7^ activated Th1 cells at DG10.5 and DG12.5 (*n* = 24)	MSCs suspended in 100 mL of PBS (106 cells/100 mL) injected on DG 11.5 and DG 13.5	Nothing	Systolic DG14: 135**	Systolic DG14: 182	DG14: 750 mg/L	DG14: 750 mg/L
Wang, 2016 [33]	Pregnant rats treated with 1 μg/kg LPS solution (n = 21)	2 × 10^6^ MSCs per rat	Nothing	Systolic DG12: 116.27 ± 0.61 DG15: 125.08 + 3.42 DG18: 124.29 ± 3.71*	Systolic DG12: 115.65 ± 1.15 DG15: 127.41 ± 3.25 DG18: 139.50 ± 2.38	DG13: 3.10 ± 0.71 mol/Ln DG16: 3.81 ± 0.70 mol/L DG19: 4.54 ± 0.64 mol/L*	DG13: 3.06 ± 0.50 mg DG16: 4.55 ± 0.57mgn DG19: 5.56 ± 1.22 mg

RED: Approximate value obtained by graph extrapolation

*DG* day of gestation, *LPS* Lipopolysaccharide, *PBS* phosphate buffered saline

*Statistically significant at *p* < 0.05

**Statistically significant at p < 0.01

### Meta-analyses

A meta-analysis was done amongst the studies that used AT as the treatment. A forest plot with a pooled effect was done to show the results, with the effect size synthesized using a fixed effects model. Meta-analyses for studies using A1M or MSCs were not feasible due to limited number of studies as well as varying patient populations and outcome reporting amongst the studies.

**Fig. 2:**
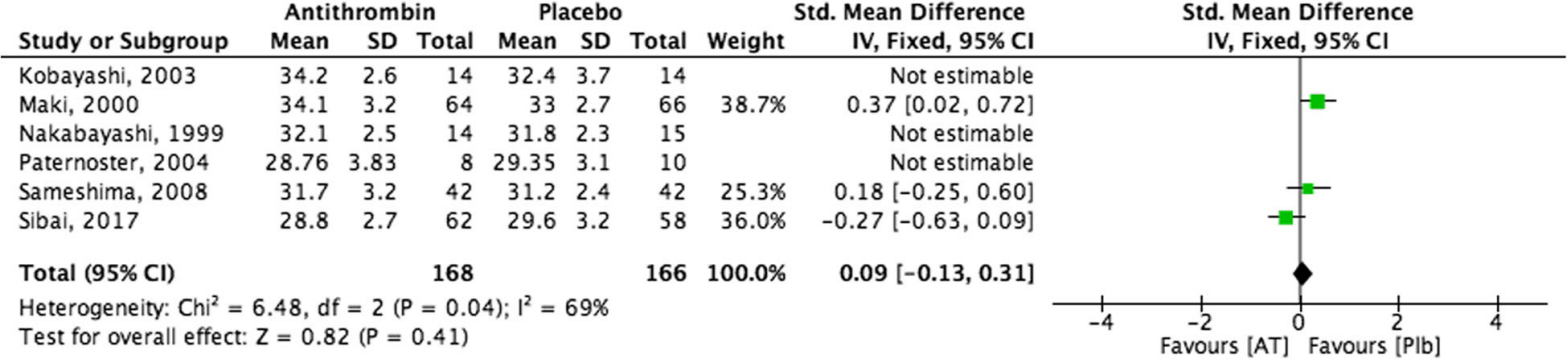
AT as a potential therapy for PE, Outcome: Gestational age at delivery

Figure 2 demonstrates the results of pooling RCT data on AT therapy versus placebo in extending gestational age at delivery in women with PE. The pooled effect and 95% confidence interval can be found at the bottom of Fig. 2, in the same line as “Total”. In the right panel of Fig. 2, the cumulative meta-analysis is graphically displayed. The meta-analysis demonstrates that the desired outcome (increased gestational age at delivery) was not favoured in the AT treated group compared with the placebo treated group. However, these results are not significant.

**Fig. 3:**
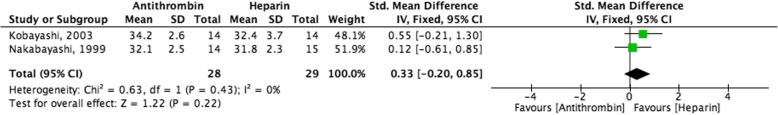
AT as a potential therapy for PE, Outcome: Gestational age at delivery in patients treated with AT and heparin, versus patients treated with heparin alone

Figure 3 demonstrates the results of pooling RCT data on AT plus heparin therapy versus heparin alone in extending gestational age at delivery in women with PE. The pooled effect and 95% confidence interval can be found at the bottom of Fig. 3, in the same line as “Total”. In the right panel of Fig. 3, the cumulative meta-analysis is graphically displayed. The meta-analysis demonstrates that the desired outcome (increased gestational age at delivery) was not favoured in the AT and heparin treated group compared with the group treated with heparin alone. However, these results are not significant.

## Discussion

The treatment of PE is more challenging than its prevention. The literature demonstrated that the pathology of PE cannot be completely reversed or terminated. Therefore, current methods of treatment are used to decrease the rate of advancement of the pathological process in order to prolong pregnancy. The current methods used to treat PE include treating hypertension, aspirin and control of blood sugar and renal function [36].

From a review by El Sayed published in 2017 [36], the following medications have proven safe and effective in prolonging pregnancy in women with PE: esomeprazole, which potently decreases soluble fms-like tyrosine kinase 1 and soluble endoglin secretion from placenta and endothelial cells, and has biological actions to mitigate endothelial dysfunction and oxidative stress [37]; metformin, an inhibitor of hypoxic inducible factor-1a [38]; sildenafil, a vasodilator [39]; curcumin, an anti-Toll-like receptor-4 [40]; and, hydroxyl-chloroquine, an antagonist of tumour necrosis factor-a [41]. However, these drugs used individually in PE have only been able to prolong pregnancy for 2–4 days, albeit a sufficient time frame to allow for a single course of steroid therapy which has been shown to improve fetal outcomes [42]. Although, the efficacy of using multiple medications is unknown, and could prove to prolong pregnancy even further in these women, there still remains an unmet need for a successful treatment option.

The current study evaluated the literature for evidence relating to three potential new treatment options for PE: AT, A1M, and MSCs. A systematic review of the literature provided limited data for these treatment options, with clinical data only being available for AT. Unfortunately, a meta-analysis that included six clinical studies comparing AT and placebo in women with PE demonstrated no difference between the two study groups for gestational age at delivery. Furthermore, when data from two studies comparing AT with heparin versus heparin alone were combined, no difference in gestational age at delivery was observed between the two study groups. Regarding A1M, data from the animal studies were variable with benefits observed for some but not all outcomes. For MSCs, benefits were observed in a majority of outcomes with a significant decrease in blood pressure shown in all three animal studies, and a significant decrease in proteinuria demonstrated in two out of three of the animal studies.

At the time of finalizing this manuscript (August 2018) a search of the literature was performed using relevant key words to see if any new publications existed in the topic area. Two studies were identified [43, 44]. The first study evaluated the biological function of MSCs for the treatment of angiotensin receptor agonistic autoantibody-induced hypertension during pregnancy in a rat model [43]. The study found that MSCs ameliorated induced hypertension, intrauterine growth retardation, kidney damage, and spiral artery remodeling impairment [43]. The second study demonstrated that A1M had a protective effect on kidneys in a mouse model of acute kidney injury [44]. The same study also constructed a novel recombinant A1M with superior solubility and stability, and suggested it was a better drug candidate for treating acute kidney injury and preeclampsia [44]. Both these studies, although not retrieved during the systematic review process, support the benefits of A1M and MSCs that we observed in our findings.

This study was strengthened by a rigorous search strategy. However, our results and the strength of our conclusions are limited by the number of relevant studies [16], and the number of relevant human studies [7]. In addition, evaluations were inconsistent and/or lacking across studies. For example, no human studies reported on normal-term delivery but instead reported on gestational age at delivery or pregnancy prolongation. Furthermore, no human studies reported on all four outcomes of interest in the same study (blood pressure, proteinuria, pregnancy stage, and biomarkers), with most human studies reporting on just a few outcomes. In fact, biomarkers were only evaluated in the ex vivo studies and only one of the human studies reported on blood pressure. Lastly, numerical values for the outcomes of interest were not always available which resulted in values being extrapolated from graphs which introduced a percent of error to these data.

## Conclusions

In conclusion, the results of this review are limited by the quantity and quality of evidence available but are supported by a thorough systematic search and review of the literature. According to the current systematic review, at this time, it appears that clinical data do not support using AT as a treatment option for PE. Regarding A1M and MSCs, animal and ex vivo studies identified in this review provide data to support pilot clinical studies of both of these agents in women with PE. However, our review suggests that the current experimental data favors MSCs over A1M.

## Additional file

Additional file 1: Search Strategy. Additional details of the primary search strategy developed using Ovid MEDLINE and EMBASE (DOCX 72 kb)

## Abbreviations

A1M: alpha-1-microglobulin
AT: antithrombin
BP: blood pressure
HbF: fetal hemoglobin
HELLP: Hemolysis, Elevated Liver enzymes, Low Platelet count
miRNA: microRNA
MSC: mesenchymal stem cell.
PE: preeclampsia
RCT: randomized controlled trial
