# Supplementary material for: Potential biological therapies for severe preeclampsia: a systematic review and meta-analysis
Source: BMC Pregnancy Childbirth. 2019 May 9;19:163. doi: 10.1186/s12884-019-2268-9 (PMC6509856; doi:10.1186/s12884-019-2268-9)
Supplement: Supplementary file 1 — Search Strategy. Additional details of the primary search strategy developed using Ovid MEDLINE and EMBASE (DOCX 72 kb) [file 12884_2019_2268_MOESM1_ESM.docx]

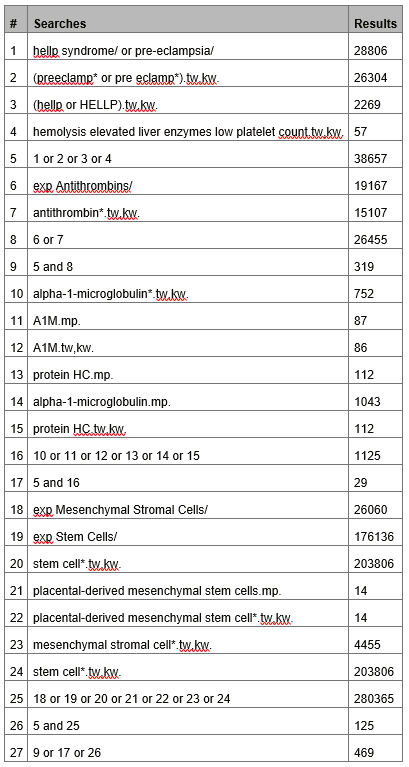

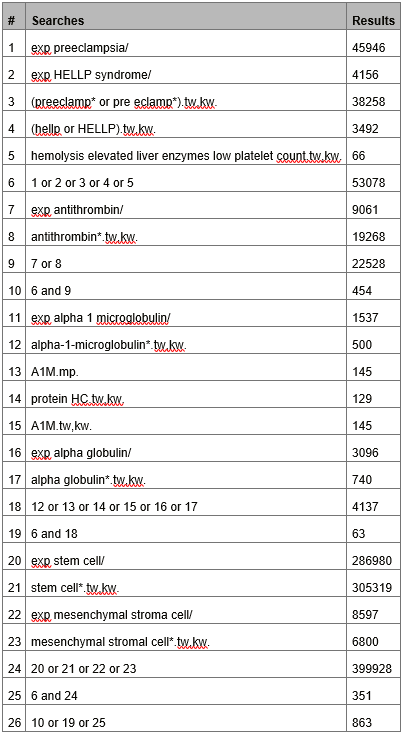


*Search strategy using Ovid MEDLINE (left) and EMBASE (right)*

The primary search strategy was developed using Ovid MEDLINE and EMBASE. Relevant articles were gathered using medical subject headings (MeSH) and text words (tw).

Databases: Ovid MEDLINE and EMBASE <Inception-May 30, 2017>

Search Date: May 30, 2017
